# Household

**Published:** 1889-12

**Authors:** 


### HOUSEHOLD.

Remove Broken Eggs.—Egg shells should never be given to hens, as they will learn the vice of egg-eating thereby, When an egg is broken in the nest or yard, it should be removed as soon as discovered. A hen seldom begins to eat egg shells until she finds one broken by accident, or until she becomes accustomed to egg shells that may be thrown into the yard.

Hanging Baskets, well filled with healthy, growing plants, are the finest objects in the window garden. For this purpose the dwarf nasturtium is a fitting subject. Fill your basket with light, gravelly soil, put in a few seeds of the desired kind, and keep well watered, and by the time the basket is wanted for its position it will be well filled, and will remain an object of beauty the entire winter.

Breakfast Cakes.—One and a half cups of Indian meal, one and a half cups of flour, half a cup of sugar, butter, a teaspoonful of soda, milk, one egg ; stir cream of tartar in the flour and dissolve the soda in a little cold water ; mix all this quite soft with milk; bake in shallow pans. To be eaten hot with butter.

Lady Cake.—One pound flour, five eggs, one pound sugar, tumbler of milk, half pound butter, teaspoonful soda, juice and rind of a lemon, and twelve bitter almonds, blanched and pounded. Bake it thin, in three small sheets.

Mount Pleasant Cake.—Two and a half cups sugar, five cups of flour, one cup butter, one cup sour milk, one teaspoon yeast powder, four eggs. Mace, citron and currants to taste.

Virginia Corn Bread.—Break into a bowl two eggs, adding a teaspoonful of soda and twice as much salt. Beat well. Stir into this mixture a pint of sour milk or buttermilk, then add a pint of corn meal and stir to a smooth batter. Put into a small baking pan a piece of lard about the size of an egg; heat it to a frying heat on the top of the stove, pour in the batter, place the pan in the oven and bake quickly.

Maccaroni Soup.—Take four ounces maccaroni,break into small pieces,and simmer gently for a quarter of an hour in one pint of water, then add a piece of butter the size of a small nutmeg, pepper, salt, and 1½ pints of stock. A teaspoonfu of chopped parsley or dried herbs can be added for flavoring. Simmer another half hour and serve.

Muffins.—A home-made and well-tried recipe for muffins is one pint of sweet milk, a piece of butter the size of an egg (or a little smaller), salt, one egg, three heaped teaspoonfuls of baking powder, and flour enough to make stiff enough to drop nicely in pans.

Crumpets.—Take two lbs. of flour made into dough with warm milk and water, adding a little salt,three well-beaten eggs,and three teaspoonfuls of yeast all mixed to the consistence of a thick batter. After standing before the fire for a short time to rise, pour into buttered tins, and bake slowly to a fine yellow.

